# Supplementary material for: Prognostic factors in clinical stage IIIA small cell lung cancer: An analysis of a population-based cancer registry in Taiwan
Source: PLoS One. 2024 Aug 29;19(8):e0297399. doi: 10.1371/journal.pone.0297399 (PMC11361662; doi:10.1371/journal.pone.0297399)
Supplement: Attachment — Submitted filename: response to reviewer.docx [file pone.0297399.s001.docx]

Dear Editor and Reviewers

Thank you for spending your valuable time to review our paper. Fortunately, this paper could be submitted to PLOS one. We were grateful to Editors and Reviewers to review this paper and make some useful recommendation. That made this paper more valuable. It was our honor to have an opportunity to publish in PLOS one. We have considered the review’s comment and replied

them as below.

Review Comments to the Author

Reviewer #1: Although the study potentially suggests that surgery is better for stage 3A small cell lung cancer, I have some concerns;
1- I think there is an important bias because those who are younger, have better performance, and have a more clinically limited disease are probably selected for the operation

Thanks for your suggestion. Although the NCCN guidelines do not recommend

surgery as an standard treatment for stage IIIA small cell lung cancer. In our study, we introduced a new perspective that surgery should be considered as one of the alternative treatment. Furthermore, we have observed that young age, lower Charlson Comorbidity Index and clinically limited stage , the 5-year survival rate tends to be better(detailed data have been included in table. 1:

Patient demographic data and univariate survival analysis)

2- Whether there is a discrepancy between post-op pathology and pre-op clinical staging in patients who have undergone surgery seems to be a factor that may affect the study results

Thanks for your suggestion. We also consider that post-op pathology and pre-op clinical staging is an important factor may affect the 5-years overall. We will arrange further research that post-op pathology and pre-op clinical staging in patients whether affect the overall survival rate.

3- It is not stated in what proportion of patients PET-CT was used for staging, and whether bone scintigraphy was performed in addition to tomography for staging in those who did not use PET-CT. This raises questions about how many patients are accurately staged.
Despite all this, I see the study as an encouraging effort for surgery in selected patients with stage 3A small cell lung cancer..

Thanks for your suggestion. We also understand the importance of PET-CT and bone scan in accurately staged. The Taiwan National Health Insurance system in taiwan provides full coverage for PET-CT and bone scans. Therefore, overall, the clinical staging of lung cancer patients is relatively accurate.

Reviewer #2: In this current research, the authors aimed to investigate prognostic factors and the impacts of different treatment methods on overall survival for stage IIIa small cell lung cancer in Taiwan.

Comments:
1. Please try restructuring the introduction by dividing it into paragraphs.

Thanks for your suggestion, we will reorganize the introduction as requested, dividing it into paragraphs.

2. "Variables with a p-value less than 0.10 in the univariate analysis were entered into the multivariate analysis,..."
Please give a reference for this sentence. Why did you include the .10 ones in the multivariable analysis?

Bursa et al. (Source Code Biol Med. 2008; 3: 17) established criteria for the purposeful selection of covariates, setting the variable entry threshold at 0.25 and the variable retention threshold at 0.10 to minimize discrepancies due to non-comparable parameters. The purposeful selection of covariates aims to minimize discrepancies arising from non-comparable parameters. However, we did not employ the purposeful selection of covariates. Instead, we revised the statistical statement "Variables with a p-value less than 0.10 in the univariate analysis were entered into the multivariate analysis..." to "The selected covariates, including age, sex, Charlson Comorbidity Index (CCI) score, histology grade, clinical stage, and treatment modality, were associated with cancer prognosis. The univariate analysis included all selected clinicopathological factors. The background knowledge criteria (refer to Biom J. 2018 May; 60(3): 431–449) were applied for selecting all covariates in the multivariable analysis."

3. In some places, the expression "men" and, in others, "male" were used. Please provide a standard.

Thanks for your valuable suggestion. We will correct the grammar and inconsistency in the article.

4. Regarding the issue I mentioned in the second comment, it seems that multivariable analyses of all variables were made in the tables?

We revised the statistical statement "Variables with a p-value less than 0.10 in the univariate analysis were entered into the multivariate analysis..." to "The selected covariates, including age, sex, Charlson Comorbidity Index (CCI) score, histology grade, clinical stage, and treatment modality, were associated with cancer prognosis. The univariate analysis included all selected clinicopathological factors. The background knowledge criteria (refer to Biom J. 2018 May; 60(3): 431–449) were applied for selecting all covariates in the multivariable analysis."

5. There is no need to give values such as p values and hazard ratios in the discussion.

Thanks for your valuable suggestion. We will remove any redundant descriptions to make the article more concise.

6. Please do not use further abbreviations after stating the abbreviations. E.g. Charlson Comorbidity Index (CCI) score

Thanks for your valuable suggestion. We will correct further abbreviations

7. Express the conclusion section more clearly. Why is future research needed? Also provide a perspective for future research.

Thanks for your valuable suggestion. In our study, surgery should be considered as one of the alternative treatment in stage IIIA small cell lung cancer. We will delete the below paragraph (Therefore, further investigation is required to gain more profound insights into these prognostic factors). The future research we will explore the feasibility of surgery in patients with stage IIIA lung cancer and include more relevant data. E.g. post-op pathology and pre-op clinical staging

8. There were lots of grammatical and spelling errors throughout the manuscript. The authors should meticulously revise their manuscript regarding typos and grammar. A native speaker's revisions would be beneficial.

Thanks for your valuable suggestion, we will correct any grammatical and spelling errors and seek revisions from a native speaker.

Although the manual provides valuable information, it needs to be reviewed meticulously.

Reviewer #3: The authors are doing survival analysis based on clinical features. This paper is statistical analysis report of Taiwan Cancer Registry. Surgery and stages are important in prognosis and they validated that in new data set.

Thank you for taking the time to review our paper. We are grateful to the reviewers for their valuable feedback and recommendations, which will help to improve the clarity and conciseness of our article.
